# Contribution of a standardized Neuropsychomotor assessment (NP-MOT battery) associated to the WISC-V scale in order to better understand a dysgraphia impairment highlighted by a heterogeneous IQ profile in a High Intellectual Potential child

**DOI:** 10.1192/j.eurpsy.2023.1600

**Published:** 2023-07-19

**Authors:** L. Vaivre-Douret

**Affiliations:** 1 Faculty of Health, Department of Medicine Paris Descartes, University of Paris Cité, Paris; 2 INSERM Unit 1018-CESP, Faculty of Medicine, University of Paris-Saclay, UVSQ, Villejuif; 3 Chair in Clinical Neurodeveloppmental Phenotyping, Institut Universitaire de France (IUF); 4 Department of Endocrinology, IMAGINE Institute of Necker-Enfants Malades hospital; 5 Department of Child Psychiatry, Necker-Enfants Malades Hospital, AP-HP.Centre, Paris, France

## Abstract

**Introduction:**

Many research studies and clinicians consider a heterogeneous IQ profile as a specific developmental characteristic to High Intellectual Potential (HIP), despite difficulties in handwriting. We propose to illustrate by a case study, the interest of supplementing a scale IQ with a standardized neuropsychomotor assessment.

**Objectives:**

We report the complex evaluation of a 8.5 years old boy with an IQ = 137, assessed HIP with a heterogeneous profile at the WISC-V but presenting a clumsiness with a dysgraphia (using the right hand) and difficulties in geometry. These disorders have been attributed by a psychologist to a fast thinking that can impact his graphomotor gesture. However, we aimed to better understand the gap between some IQ index scores.

**Methods:**

We have conducted a complete standardized assessment of developmental neuropsychomotor functions (NP-MOT battery, Vaivre-Douret. Digital Ed Neuralix®, 2021; https://neuralix-editions.com/) with age-related normative data, and of neuropsychological functions, in addition an oculomotor examination (Eye-tracking).

**Results:**

The IQ index scores are: VCI = 155, VSI = 108, FRI = 137, WMI = 138, PSI = 92. We found with the NP-MOT battery, a left-handed laterality and at the muscular tone examination, a motor dysfunction of the pyramidal tract on the left body distal side (mild spasticity) and oculomotor disorders of the visual pursuits, associated to visual-spatial motor and visual motor integration impairments.

**Conclusions:**

It is a neurologically right-handed child because he can not effectively use his left hand to correctly write but he is not so good with the right hand to write. Moreover, he presents a visual-spatial motor subtype (< -2 SD) of developmental coordination disorder (DCD according the DSM-5) with oculomotor abnormalities, explaining his clumsiness and dysgraphia, and his difficulties in geometry. Thus, the subtests that make up VSI and PSI highlight a motor component (graphomotor, oculomotor, visuomotor) that should be analyzed in the light of additional neuropsychological and normed assessments of developmental neuropsychomotor functions.

Comorbidity of neurological and motor coordination disorders do not spare the child with a high intellectual potential, despite his high mental abilities helping him to compensate. It is important to complete the WISC-V scale by other investigations, particularly in the motor field, to explain the heterogeneity of the IQ profile with scattered index scales.

**Disclosure of Interest:**

None Declared

